# The Impact of School Tobacco Policies on Student Smoking in Washington State, United States and Victoria, Australia

**DOI:** 10.3390/ijerph7030698

**Published:** 2010-02-26

**Authors:** Tracy J Evans-Whipp, Lyndal Bond, Obioha C Ukoumunne, John W Toumbourou, Richard F Catalano

**Affiliations:** 1 Centre for Adolescent Health, Murdoch Childrens Research Institute, 2 Gatehouse Street, Parkville, Vic, 3052, Australia; 2 Social and Public Health Sciences Unit, Medical Research Council, 4 Lilybank Gardens, Glasgow, G12 8RZ, UK; E-Mail: l.bond@sphsu.mrc.ac.uk; 3 Clinical and Epidemiological Biostatistics Unit, Murdoch Childrens Research Institute, Royal Children’s Hospital, Parkville, Vic, 3052, Australia; E-Mail: obioha.ukoumunne@mcri.edu.au; 4 School of Psychology, Deakin University, Geelong Waterfront Campus, Geelong Vic, 3217, Australia; E-Mail: john.toumbourou@deakin.edu.au; 5 Social Development Research Group, School of Social Work, University of Washington, 9725 3rd Avenue NE, Seattle, WA 98115, USA; E-Mail: catalano@u.washington.edu

**Keywords:** schools, tobacco policy, tobacco smoking

## Abstract

This paper measures tobacco polices in statewide representative samples of secondary and mixed schools in Victoria, Australia and Washington, US (N = 3,466 students from 285 schools) and tests their association with student smoking. Results from confounder-adjusted random effects (multi-level) regression models revealed that the odds of student perception of peer smoking on school grounds are decreased in schools that have strict enforcement of policy (odds ratio (OR) = 0.45; 95% CI: 0.25 to 0.82; p = 0.009). There was no clear evidence in this study that a comprehensive smoking ban, harsh penalties, remedial penalties, harm minimization policy or abstinence policy impact on any of the smoking outcomes.

## 1. Introduction

The number of young people smoking in Australia and the United States (US) has decreased steadily since the mid-1990s [1,2]. Given the numerous and well-documented adverse effects of smoking [3], however, and the fact that tobacco use remains the leading cause of preventable death in the United States and Australia [4], youth smoking prevention remains a high public health priority.

Schools have been considered ideal sites in which to deliver tobacco prevention programs since they capture the majority of youth across a large time period which includes the ages most young people initiate smoking. In response, schools have implemented curriculum-based smoking prevention programs or “drug education” in some form or other since the 1980s. In addition to prevention curricula, the majority of secondary schools in the US and Australia develop and implement tobacco policies that describe expectations for tobacco use in the school environment and detail the consequences for those found violating policy restrictions [5]. Smoke-free schools, in which all staff and visitors are banned from smoking on school grounds and at school events, serve to reduce students’ exposure to tobacco smoke and remove smoking role models from students’ daily school life.

Research into the effectiveness of school tobacco policies has been conducted over the last fifteen years but methodological issues have precluded any definitive conclusions as to the impact of school policies on student smoking and what policy components are important [5,6]. This is unsurprising given that the studies conducted to date have measured school policy in different ways (coding of written documents, school administrator reports or student reports) and used different measures of smoking (daily, less than daily, current, smoking susceptibility and stage of smoking uptake) as well as different groups of students (ages, ethnicity, location, *etc.*). There are also analytic issues to consider that earlier studies failed to take into account. The first of these is the appropriate use of random effects (or “multi-level”) modeling to account for the commonly used sampling design in which students are clustered within schools. Since students in the same school are more likely to be similar their responses are not independent and bias might occur. The second issue relates to the proper use of potential confounders in analyses. Aveyard and colleagues have argued that it is inappropriate to control for certain known pupil-level contributors to student smoking (such as attitudes towards smoking and best friend’s smoking) since these might represent the mechanism by which school tobacco policy influences student behavior and to do so would reduce the ability to detect policy effects [6]. Despite these challenges there is some evidence for important domains of effect such as strongly enforced policies and comprehensive smoking bans.

Comprehensive smoking bans represent the most restrictive form of smoking policy in that they prohibit any smoking on school grounds by students, staff and visitors thereby serving to establish a “smoke free school”. Whilst some earlier studies have reported inverse relationships between policy comprehensiveness and student tobacco use [7], more recent studies have failed to find an effect [8,9]. Despite the ambivalent empirical data, most policy guidelines emphasize the importance of removing smoking role models from students’ view in order to foster a non-smoking norm. In addition to the details of policy content, researchers have investigated the consistency with which schools enforce policy responses (regardless of the type of sanction imposed) to student smokers and have shown this to be an important component of policy effectiveness [7,9–11]. It is perhaps unsurprising that conspicuous actions taken by the school in response to policy violations rather than the mere presence of policies themselves are what influences student perceptions of policy and behaviors.

The specific actions taken in response to students who violate school smoking policies are likely to be an important component of their impact. Whilst nearly all secondary schools ban student smoking the events following a policy violation may vary considerably and this demonstrates how schools can view and use their tobacco policies in differing ways. On the one hand schools might regard student smoking solely as a disciplinary matter and issue harsh consequences (such as expulsion or out of school suspension) to policy violators, or alternatively the school might view student smoking more as a health issue and offer remedial consequences such as counseling, cessation or education programs whilst working to maintain the student in the school setting. In many cases a combination of these two approaches will be employed. Few studies have examined the impact of this variation on student smoking.

The terms “abstinence” and “harm minimization” often occur in the context of drug and alcohol policies. Abstinence refers to the goal of no drug use and in the context of school tobacco policies implies that schools promote a non-smoking ideal. Harm minimization, as a principle, is more accepting of the fact that a number of young people will experiment with tobacco and, whilst promoting abstinence as the means of least harm from tobacco, also aims to work with students who have experimented with smoking and are in the early stages of becoming a regular smoker. Abstinence-focused approaches would be more likely to apply punishment to this group of students since they have violated abstinence policies.

This paper reports results from the International Youth Development Study (IYDS), a longitudinal study of a range of adolescent behaviors in statewide representative samples of school students in Washington State, US and Victoria, Australia. These two states provide interesting samples to study school policy effects since they share many demographic, economic and ethnic similarities but differ in their frameworks for addressing youth drug use. Australian policies have explicitly endorsed the goal of harm minimization whereas in the US encouraging abstinence and delaying first use remain important prevention priorities. In addition to a comprehensive student survey the IYDS collected detailed information on the drug policies of each of the sampled schools via a questionnaire administered to the Principal or nominated staff member. A review of schools’ reports of their drug policies in Wave 1 of the Study confirmed that schools in each state implemented policies consistent with national policy frameworks [12]. Thus the IYDS sample contains a high degree of variation on its measures of school tobacco policy. This paper uses data collected in Wave 2 of the Study (2003) to investigate whether exposure to particular types of school tobacco policy is associated with differential risk of student smoking.

## 2. Methods

### 2.1. Participants and Procedures

Cross-sectional data used in this analysis were collected during the second year (2003) of the International Youth Development Study (IYDS), a longitudinal research study of adolescent substance use patterns in Washington State and Victoria. Procedures for the IYDS sampling, school administrator survey and student survey have been described previously [12–14]. Briefly, the total sample comprises statewide representative samples of students in Victoria, Australia (N = 2,884) and Washington State, U.S (N = 2,885) from 3 age cohorts (Grade 5, Year 7 and Year 9). In Wave 2, 5,692 students (99% retention rate in both states) in grades 6, 8 and 10 completed a student questionnaire in a class-based setting (a small percentage of students were interviewed by telephone) and a selected school staff member from each participating school completed a school administrator mail survey. Since only one third of Victoria primary schools (up to Grade 6) reported having a written drug policy [12] the current study does not use data collected from primary/elementary schools or the 1,852 Grade 6 students attending them. Honesty criteria (described below) were used to remove a further 40 students from the sample. Another 393 students were dropped from the sample because their school did not complete the School Administrator Survey. The final sample analyzed comprised 3,466 students from 285 schools.

### 2.2. Measures

#### Student-level outcome variables

The measure of current tobacco use was a binary indicator of self-reported smoking. Students were asked, “How frequently have you smoked cigarettes in the past 30 days?” Response options ranged from ‘not at all’ to ‘40 or more cigarettes a day’ on an 8-point scale. Students responding ‘not at all’ were assigned to a ‘non-smoker’ group and those responding ‘less than one a day’ or more were assigned to a ‘current smoker’ group.

To measure daily smoking students were asked “Have you smoked in the past year?” Those responding ‘almost every day or everyday’ were classified as daily smokers and those responding ‘never’, ‘once or twice’, ‘once in a while but not regularly’, ‘regularly, but less than every day’ as non-daily smokers.

Students were asked to rate student smoking on school grounds by their agreement to the following item: “many students smoke on school grounds without getting caught”. The response set was rated on a four point scale from ‘YES!’, through ‘yes’ and ‘no’ to ‘NO!’. Those responding ‘YES!’ or ‘yes’ were classified as perceiving that many students smoke on school grounds and those responding ‘NO!’ or ‘no’ were classified otherwise.

#### School-level exposure variables

Schools were rated as possessing a comprehensive smoking ban if they responded yes to all of the following 4 questions: Are your teachers and staff covered by a policy that prohibits tobacco use in the school building?; …on school grounds?; …during school related activities where students are present?; Are visitors to the school covered by a policy that prohibits tobacco use on school grounds?

To index policy orientation toward abstinence and harm minimization principles, administrators were asked to indicate the degree to which the following two statements described their school: “School policies emphasize total abstinence from drug use” and “School policies are based on the assumption that most youth will experiment with drugs”, respectively. Response options ranged from ‘not at all’ (1) to ‘a lot’ (5).

School administrators were asked to rate the enforcement of their school’s substance use policy on a 4-point scale. Policy was rated as strictly enforced (‘very strict’ response) or not strictly enforced (‘moderately strictly’, ‘not very strictly’ or ‘not at all strictly’ responses).

School administrator reports of how often students caught using or possessing tobacco on school grounds or at school events received a particular response were used to classify ‘harsh’ and ‘remedial’ punishments for tobacco policy violations. For a harsh response, school administrators had to respond ‘almost always or always’ to one or more of the following three responses: expulsion, calling the police and out of school suspension. For a remedial response school administrators had to respond ‘almost always or always’ to one or more of the following three responses: referred to a school counselor or nurse, recommended to participate in an assistance, education, or cessation program or required to participate in an assistance, education, or cessation program.

#### Honesty

A single measure of honesty was calculated based on student responses to 3 survey items including use of a fictional drug as described in greater detail in [15]. Forty students recognized as dishonest were removed from this analysis.

#### Family socioeconomic status (SES)

State, gender, age and family SES were investigated as potential confounders of the relationships between school policies and smoking outcomes. A single composite measure of family SES was calculated from responses to questions on maternal and paternal education status and family income provided in a separate telephone interview with a parent/guardian of each student in Wave 1 of the study as described in [16].

### 2.3. Statistical Analysis

The school policy, smoking outcome and potential confounding variables were summarized, for each state separately, using means and standard deviations for symmetrical variables, medians and interquartile ranges for non-symmetrical quantitative variables and numbers and percentages for categorical variables. The Chi-squared test was used to compare the categorical school policy and student smoking variables between states and independent samples *t* tests were used to compare means of abstinence and harm minimization measures between states. Random effects logistic regression using maximum likelihood was used to estimate the increase or decrease in odds of each smoking outcome for each specific school policy component. These analyses modeled the random effects at the school (cluster) level. The model was estimated using maximum likelihood with the adaptive Gauss-Hermite quadrature algorithm used to carry out the numerical integration required to approximate the likelihood [17]. Both unadjusted analyses and analyses adjusted for the potential confounding variables state, gender, age and family SES were implemented. Tests of interaction were used to assess evidence that the effects of school policy on smoking outcomes differed between the two states and between males and females. Data analysis was performed using Stata version 10 with the *xtlogit* command used to run the random effects logistic regression [18].

## 3. Results

Table 1 presents the sample characteristics for Washington and Victoria. Students’ self-reported smoking status showed that smoking is more prevalent amongst students in Victoria than in Washington with around twice as many Victorian students reporting smoking in the past 30 days than Washington students (p < 0.001) and over three times as many reporting daily smoking in the past year (p < 0.001). Reports of student smoking on school grounds (without getting caught) were also higher in the Victorian sample (63.8% *versus* 34.9%, p < 0.001).

The number and proportion of schools reporting use of various policy types are also given in Table 1. More Washington schools used comprehensive smoking bans (p < 0.001), harsh (p < 0.001) and remedial penalties (p = 0.02), and strictly enforced policy (p < 0.001) as indicated by school administrators in the school survey. Washington schools reported that their policies placed significantly more emphasis on abstinence principles than Victorian schools (p < 0.001) whereas Victorian schools scored more highly on the harm minimization measure (p < 0.001). Washington students were more likely to be older and have a higher family SES score than Victorian students.

The random effects logistic regression models used to investigate associations between aspects of school tobacco policy and student smoking were conducted for the combined Washington and Victoria datasets as tests of interaction provided no strong evidence of differential effects between states. The unadjusted and adjusted odds ratios for associations with current smoking are given in Table 2. There was no strong evidence of changes in the odds of student smoking in the presence of a comprehensive smoking ban or with the use of remedial penalties. School administrator reports of the use of harsh penalties and strict smoking policy enforcement were associated with a reduction in the odds of current student smoking although the magnitude of these effects was reduced in the adjusted analyses. Policies based on abstinence principles were associated with a decrease in the likelihood of current student smoking (OR = 0.84; 95% CI 0.74 to 0.95; p = 0.006) whereas policies based on harm minimization principles were associated with an increase in the likelihood of current student smoking (OR = 1.19 95% CI 1.06 to 1.34; p = 0.002) although again there was no strong evidence for these effects in the adjusted models.

In addition to looking at the impact of school policy components on current smokers we investigated the associations with policy on more established smokers as defined by having smoked on an almost daily or daily basis over the past year. The results (shown in Table 3) were similar to those observed with current smoking: no strong evidence of changes in the odds of student smoking in the presence of a comprehensive smoking ban or the use of harsh or remedial penalties or abstinence or harm minimization based policies were detected. Strict policy enforcement and abstinence based policies were associated with lower odds ratios and harm minimization based policies were associated with increases in odds ratios for daily smoking in unadjusted analyses, but after adjusting for confounders there was no evidence at the 5% level for these effects.

Results of associations between school policy components and perceptions of peer school smoking are reported in Table 4. Similar to student self-reported smoking, there was no detectable influence of a comprehensive school smoking ban or use of remedial penalties. Strictly enforced policies were associated with a greater than 2-fold reduction in the odds of perceptions of peer smoking at school after adjusting for confounders (OR = 0.45 95% CI 0.25 to 0.82). Harsh penalties and polices based on abstinence principles were also related to less perception of student smoking on school grounds but the strength of these relationships weakened after adjustment. Policies based on harm minimization principles were associated with an increase in perceptions of student smoking on school grounds although again the magnitude of this effect was reduced in the adjusted model.

## 4. Discussion

Schools develop and implement smoking policies in order to protect students and school staff from the harmful effects of environmental tobacco exposure and to convey the message that student smoking is not acceptable. Whether school smoking policies are an effective tool for preventing youth smoking remains to be proven. This paper investigated the associations between various types of school tobacco policy and student smoking as well as student perceived smoking on school grounds in two states with differing drug policy backgrounds.

Interestingly, there was no strong evidence in this study of an association between a comprehensive smoking ban and student smoking and so the issue of whether school smoking bans reduce student smoking remains equivocal. In an earlier UK study [7], strong/comprehensive anti-smoking policies (covering all members of the school community and visitors) were associated with lower likelihood of student daily smoking. It is notable that in that study, the rates of student smoking were considerably higher than in the current study (daily smoking was 22.9% for girls and 18.2% for boys *versus* 5.5% for girls and 3.8% for boys in the current study). A more recent study of secondary schools in Germany [19] also found that student smoking bans were associated with lower rates of student current smoking. Again, the rates of self-reported student smoking in this study were considerably higher than in the current study (23% students reporting current smoking *versus* 14% in the current study) thereby raising the possibility that there is a ceiling effect for the impact of comprehensive smoking bans in schools that has been reached in the current study populations. Adult smoking rates are higher in Germany and the UK than in Washington and Victoria which is perhaps indicative of Washington State and Victoria being further ahead of the UK or Germany in their efforts to reduce smoking in their populations in general. It may be increasingly difficult to detect an impact of school smoking policies on youth smoking in countries where local, state or national tobacco control laws, such as legislating smoke-free schools/tobacco free environments, have been introduced. For example, in Australia, the state government of Victoria introduced smoke-free schools legislation in 2009 [20]. Regardless of our ability to detect an effect on concurrent student smoking it could be hypothesized that the normative influence of such policies will still have an effect on young people’s attitudes towards smoking and intentions to smoke that might take years to accumulate and develop. Such factors have not been investigated in the current study and would be an important avenue for further research.

Strict enforcement of school tobacco policies was associated with a reduction in the perception of peer smoking on school grounds and although it did not remain as an independent predictor of current and daily smoking when controlling for other factors there is a suggestion that strict enforcement could be impacting on student self-reported smoking. There has been speculation that the enforcement of anti-smoking policies is of greater importance than the detailed contents of the policy such that minor improvements to content are unlikely to have a great effect on a consistently enforced policy [9]. Thus schools might be best placed to direct their efforts towards increasing students’ awareness of anti-smoking policies and making implementation efforts, such as monitoring of school grounds, more apparent.

There was no strong evidence of associations between schools’ reported use of harsh or remedial consequences and student self-reported current or daily smoking in the current study. The detected association between harsh penalties and decreased student perceptions of peer smoking at school is suggestive that such policies might reduce perceptions of peer smoking. This effect might be important because students are known to be influenced by peer smoking (especially amongst older students) at school [21–25]. It is perhaps not surprising that there was no strong evidence in this study that remedial penalties are associated with any noticeable reductions in the odds of student smoking since these approaches are not developed for use as deterrents. Rather, such approaches are employed to reduce the chances of students caught smoking continuing on a pathway to established smoking. It will be important for future research studies investigating the effects of school policy to measure possible impacts at later time-points in order to investigate the longer term effects of the use of certain responses to policy violations. Schools will benefit from having clear evidence of the impact of their harsh and remedial response options on student smoking behaviors as well as a range of other student wellbeing indicators.

This study also attempted to determine the differential impact, if any, of school administrator perceptions that their school smoking policies were underpinned by harm minimization or abstinence principles. In the unadjusted analyses we found some evidence of associations between policy type and current student smoking such that abstinence-based policies were associated with reduced odds of current and daily smoking and perceptions of student smoking on school grounds and harm minimization-based policies with increased odds of all three smoking-related outcomes. Results from our previous studies [16] suggest that country differences in administrator perceptions of policy underpinnings are also observable to students, raising speculation about the contribution of school policies to the differences in smoking rates between Washington and Victoria. Students in Washington State are less likely to smoke and are more likely to be subject to the school smoking policy components associated with reduced likelihood of smoking. The indication that policies based on harm minimization principles (reported more commonly in the Victorian schools) are associated with increases in the likelihood of student smoking and perceptions of student smoking on school grounds is of some concern and warrants further research.

The current study has a number of limitations that should be noted. Reports of school smoking policies were collected from a single respondent from each school. Although this respondent was most commonly the school Principal or another staff member deemed by the Principal to be most knowledgeable of school drug policies it is possible that this single respondent might have been misinformed or subject to response bias. Measuring school policy documentation and implementation would ideally involve review and coding of actual policy documents and on-site observations by research staff although this approach would be extremely costly and time-consuming in practice. One advantage of this study is the collection of school policy information from students as well as school personnel, and we have shown previously that these 2 sources of information are well aligned [16]. The use of self-report data for student smoking is a potential source of error but previous studies have shown students to be truthful and accurate when reporting their drug use in school surveys [26–28], and unless there was variation in reporting bias between schools the measured associations between student smoking and policy would be unchanged. The data used in this analysis were cross-sectional and so it is not possible to make temporal or causal inferences from the noted associations. It is also possible that there are unmeasured differences in the characteristics of schools or students that are confounded with school tobacco policies. There are, however, a number of key strengths to the study including use of two large state-representative samples with differing drug policy backgrounds. The study design also maximized the number of school units by selecting a single class from each school sampled in order to minimize the adverse effects of clustering at analysis.

This study has measured the degree to which aspects of school tobacco policies influence student smoking behaviors. Although there was only evidence for an impact of strict enforcement of school tobacco policy on student perceptions of smoking on school grounds this study suggests many avenues for further research. One of the first areas in which school tobacco policies might have an impact would be a reduction in the numbers of students smoking on school grounds and so future studies should incorporate a measure of self-reported smoking at school in addition to the peer use measure used in the current study. It is important to investigate the impacts of school tobacco policies on future as well as concurrent student smoking since at least part of the aim of school policies is to shape students intentions and future behaviors. Further investigation using longitudinal data from the IYDS will improve understanding of the impact of school smoking policy on student behavior by measuring student smoking and school tobacco policy over several years and will provide a stronger basis for causal inferences. Investigation of plausible mediating and moderating factors will allow us to unpack the potentially complex pathways by which policy documentation and enforcement might influence student understandings, beliefs, values and behaviors regarding tobacco use. For example, a model in which the influence of written policy and policy enforcement is mediated via student perceptions of school policy and student values and intentions towards smoking could be tested. Such studies will also investigate the differential impact of policy components on different genders and ages. Once a greater understanding of the impact of school tobacco policies on student smoking has been obtained, it might be possible to test some of the important components in a large-scale randomized controlled trial. The development of an effective policy, that works to reduce student smoking both in the immediate and the longer-term whilst taking into account other wellbeing and academic outcomes, is worthy of such efforts since policy is a relatively inexpensive and universal approach to youth smoking prevention.

## Figures and Tables

**Table 1.** Frequencies (percentages) ***a*** of outcome and predictor variables by state.

	*Washington N = 1,777 students*	*Victoria N = 1,689 students*
***Control variables^b^***		
Male	882 (49.6)	822 (48.7)
Age, mean (sd)	15.1 (1.1)	14.9 (1.0)
Family SES, median (interquartile range)	2.04 (1.9 to 2.4)	1.90 (1.5 to 2.3)
***Outcomes^a^***		
Current tobacco use	167 (9.5)	310 (18.5)
Daily tobacco use	41 (2.3)	119 (7.1)
Students perceive school smoking	615 (34.9)	1,071 (63.8)
***Predictors^c^***	*Washington N = 153 schools*	*Victoria N = 132 schools*
Comprehensive smoking ban	144 (96.0)	96 (73.3)
Harsh punishment for tobacco violations	67 (46.5)	27 (22.3)
Remedial punishment for tobacco violations	89 (61.8)	59 (48.0)
Strictly enforced policy	139 (90.9)	77 (58.8)
Abstinence policy, mean (sd)	4.68 (0.8)	3.81 (1.3)
Harm minimization policy, mean (sd)	2.29 (1.2)	3.05 (1.2)

*a* Except where otherwise indicated;

*b* Range of sample sizes were 1,653 to 1,777 for Washington and 1,576 to 1,689 for Victoria;

*c* Range of sample sizes (number of schools) were 144 to 153 for Washington and 121 to 131 for Victoria.

**Table 2.** Unadjusted and adjusted associations between student current smoking (1 or more times in past 30 days) and school level policy variables.

	**Unadjusted**			**Adjusted #**		
	**OR**	**95% CI**	**p value**	**OR**	**95% CI**	**p value**
	
***Predictor:***						
Comprehensive smoking ban	0.68	(0.44 to 1.06)	0.09	0.86	(0.59 to 1.25)	0.42
Harsh penalties	0.71	(0.50 to 1.01)	0.05	0.99	(0.73 to 1.35)	0.95
Remedial penalties	0.97	(0.71 to 1.34)	0.87	1.15	(0.88 to 1.51)	0.30
Strict enforcement	0.50	(0.36 to 0.69)	<0.001	0.78	(0.57 to 1.05)	0.10
Abstinence policy	0.84	(0.74 to 0.95)	0.006	0.93	(0.83 to 1.04)	0.20
Harm minimization policy	1.19	(1.06 to 1.34)	0.002	1.09	(0.99 to 1.21)	0.09

# Adjusted for control variables state, gender, age and family SES. Unadjusted relationships between control variables and student current smoking are: state (Washington) OR = 0.47, 95% CI 0.35 to 0.63, p < 0.001; gender (female) OR = 1.48, 95% CI 1.19 to 1.83, p < 0.001; age OR = 1.37, 95% CI 1.22 to 1.55, p < 0.001; family SES OR = 0.48, 95% CI 0.38 to 0.62, p < 0.001.

Sample sizes ranged from 3,208 to 3,421 students for unadjusted models and from 3,110 to 3,316 students for adjusted models.

**Table 3.** Unadjusted and adjusted associations between student daily smoking (smoking almost every day or every day in past year) and school level policy variables.

	**Unadjusted**			**Adjusted #**		
	**OR**	**95% CI**	**p value**	**OR**	**95% CI**	**p value**
	
***Predictor:***						
Comprehensive smoking ban	0.65	(0.33 to 1.29)	0.22	0.95	(0.53 to 1.69)	0.85
Harsh penalties	0.67	(0.38 to 1.18)	0.17	1.02	(0.62 to 1.67)	0.95
Remedial penalties	0.94	(0.56 to 1.58)	0.82	1.10	(0.72 to 1.69)	0.66
Strict enforcement	0.34	(0.20 to 0.56)	<0.001	0.70	(0.44 to 1.12)	0.14
Abstinence policy	0.79	(0.64 to 0.97)	0.03	0.95	(0.80 to 1.13)	0.56
Harm minimization policy	1.20	(0.99 to 1.45)	0.07	1.01	(0.85 to 1.20)	0.89

# Adjusted for control variables state, gender, age and family SES. Unadjusted relationships between control variables and student daily smoking are: state (Washington) OR = 0.30, 95% CI 0.18 to 0.49, p < 0.001; gender (female) OR = 1.55, 95% CI 1.09 to 2.21, p=0.01; age OR = 1.48, 95% CI 1.21 to 1.81, p < 0.001; family SES OR = 0.30, 95% CI 0.20 to 0.45, p < 0.001.

Sample sizes ranged from 3,200 to 3,413 students for unadjusted models and from 3,105 to 3,311 students for adjusted models.

**Table 4.** Unadjusted and adjusted associations between student perception of student smoking on school grounds and school level policy variables.

	**Unadjusted**			**Adjusted #**		
	**OR**	**95% CI**	**p value**	**OR**	**95% CI**	**p value**
	
***Predictor:***						
Comprehensive smoking ban	0.58	(0.26 to 1.30)	0.18	1.39	(0.67 to 2.89)	0.38
Harsh penalties	0.41	(0.22 to 0.76)	0.004	0.72	(0.42 to 1.22)	0.22
Remedial penalties	0.79	(0.44 to 1.42)	0.43	1.16	(0.70 to 1.91)	0.56
Strict enforcement	0.19	(0.10 to 0.35)	<0.001	0.45	(0.25 to 0.82)	0.009
Abstinence policy	0.69	(0.54 to 0.87)	0.002	0.89	(0.71 to 1.11)	0.31
Harm minimization policy	1.47	(1.18 to 1.83)	0.001	1.18	(0.97 to 1.43)	0.10

# Adjusted for control variables state, gender, age and family SES. Unadjusted relationships between control variables and student perception of student smoking on school grounds are: state (Washington) OR = 0.18, 95% CI 0.11 to 0.30, p < 0.001; gender (female) OR = 1.31, 95% CI 1.09 to 1.59, p = 0.005; age OR = 1.38, 95% CI 1.18 to 1.61, p < 0.001; family SES OR = 0.65, 95% CI 0.51 to 0.83, p < 0.001.

Sample sizes ranged from 3,209 to 3,422 students for unadjusted models and from 3,111 to 3,317 students for adjusted models.

## References

[1] White V Hayman J Smoking Behaviours of Australian Secondary Students in 2005 Report prepared for The Victorian Department of Human Services: Melbourne, Australia, June 2006.

[2] Johnston LD O’Malley PM Bachman JG Schulenberg JE Monitoring the Future National Survey Results on Drug Use, 1975–2006. Volume I: Secondary School Students (NIH Publication No 07-6205); NIH Publication No 07-6205; National Institute on Drug Abuse Bethesda, MD, USA 2007 699

[3] Mathers M Toumbourou JW Catalano RF Williams J Patton GC Consequences of youth tobacco use: a review of prospective behavioural studies Addiction 2006 101 948 958 16771887 10.1111/j.1360-0443.2006.01438.x

[4] Australia’s Health 2008. Cat. No. AUS 99 Australian Institute of Health and Welfare Canberra, Australia 2008

[5] Evans-Whipp T Beyers JM Lloyd S Lafazia AN Toumbourou JW Arthur MW Catalano RF A review of school drug policies and their impact on youth substance use Health Promot. Int 2004 19 227 234 15128714 10.1093/heapro/dah210

[6] Aveyard P Markham WA Cheng KK A methodological and substantive review of the evidence that schools cause pupils to smoke Soc. Sci. Med 2004 58 2253 2265 15047082 10.1016/j.socscimed.2003.08.012

[7] Moore L Roberts C Tudor-Smith C School smoking policies and smoking prevalence among adolescents: multilevel analysis of cross-sectional data from Wales Tob. Control 2001 10 117 123 11387531 10.1136/tc.10.2.117 PMC1747541

[8] Darling H Reeder AI Williams S McGee R Is there a relation between school smoking policies and youth cigarette smoking knowledge and behaviors? Health Educ. Res 2006 21 108 115 16037118 10.1093/her/cyh047

[9] Adams ML Jason LA Pokorny S Hunt Y The Relationship between School Policies and Youth Tobacco Use J. School Health 2009 79 17 23 19149781 10.1111/j.1746-1561.2008.00369.x PMC2826219

[10] Wakefield MA Chaloupka FJ Kaufman NJ Orleans CT Barker DC Ruel EE Effect of restrictions on smoking at home, at school, and in public places on teenage smoking: cross sectional study BMJ 2000 321 333 337 10926588 10.1136/bmj.321.7257.333 PMC27448

[11] Hamilton G Cross D Lower T Resnicow K Williams P School policy: what helps to reduce teenage smoking? Nicotine Tob. Res 2003 5 507 513 12959788 10.1080/1462220031000118559

[12] Beyers JM Evans-Whipp T Mathers M Toumbourou JW Catalano RF A cross-national comparison of school drug policies in Washington State, United States, and Victoria, Australia J. School Health 2005 75 134 140 15987007 10.1111/j.1746-1561.2005.00011.x

[13] Patton GC McMorris BJ Toumbourou JW Hemphill SA Donath S Catalano RF Puberty and the onset of substance use and abuse Pediatrics 2004 114 e300 e306 15342890 10.1542/peds.2003-0626-F PMC1892192

[14] McMorris BJ Hemphill SA Toumbourou JW Catalano RF Patton GC Prevalence of substance use and delinquent behavior in adolescents from Victoria, Australia and Washington State, United States Health Educ. Behav 2007 34 634 650 16740513 10.1177/1090198106286272

[15] Hemphill SA Toumbourou JW Herrenkohl TI McMorris BJ Catalano RF The effect of school suspensions and arrests on subsequent adolescent antisocial behavior in Australia and the United States J. Adolesc. Health 2006 39 736 744 17046511 10.1016/j.jadohealth.2006.05.010

[16] Evans-Whipp TJ Bond L Toumbourou JW Catalano RF School, parent, and student perspectives of school drug policies J School Health 2007 77 138 146 quiz 153-134. 17302856 10.1111/j.1746-1561.2007.00183.x

[17] Rabe-Hesketh S Skrondal A Pickles A Reliable estimation of generalized linear mixed models using adaptive quadrature Stata J 2002 2 1 21

[18] Stata Corporation Stata Statistical Software: Release 10.1 StataCorp LP College Station, TX, USA 2007

[19] Piontek D Buehler A Donath C Floeter S Rudolph U Metz K Gradl S Kroeger C School context variables and students’ smoking. Testing a mediation model through multilevel analysis Eur. Addict. Res 2008 14 53 60 18182773 10.1159/000110411

[20] Victorian Tobacco Control Strategy 2008–2013 Victorian Government Department of Human Services Melbourne, Australia 2008

[21] Leatherdale ST Cameron R Brown KS McDonald PW Senior student smoking at school, student characteristics, and smoking onset among junior students: a multilevel analysis Prev. Med 2005 40 853 859 15850887 10.1016/j.ypmed.2004.09.033

[22] Leatherdale ST Brown KS Cameron R McDonald PW Social modeling in the school environment, student characteristics, and smoking susceptibility: a multi-level analysis J. Adolesc. Health 2005 37 330 336 16182144 10.1016/j.jadohealth.2004.10.008

[23] Turner K West P Gordon J Young R Sweeting H Could the peer group explain school differences in pupil smoking rates? An exploratory study Soc. Sci. Med 2006 62 2513 2525 16364527 10.1016/j.socscimed.2005.11.017

[24] Molyneux A Lewis S Antoniak M Browne W McNeill A Godfrey C Madeley R Britton J Prospective study of the effect of exposure to other smokers in high school tutor groups on the risk of incident smoking in adolescence Am. J. Epidemiol 2004 159 127 132 14718213 10.1093/aje/kwh035

[25] Lovato CY Sabiston CM Hadd V Nykiforuk CIJ Campbell HS The impact of school smoking policies and student perceptions of enforcement on school smoking prevalence and location of smoking Health Educ. Res 2007 22 782 793 16987941 10.1093/her/cyl102

[26] Wills TA Cleary SD The validity of self-reports of smoking: Analyses by race/ethnicity in a school sample of urban adolescents Am. J. Public Health 1997 87 56 61 9065227 10.2105/ajph.87.1.56 PMC1380765

[27] Winters KC Stinchfield RD Henly GA Schwartz RH Validity of adolescent self-report of alcohol and other drug involvement. Year of Publication 1990−1991 Int. J. Addict 1990 25 1379 1395 2132719 10.3109/10826089009068469

[28] Brener ND Collins JL Kann L Warren CW Williams BI Reliability of the Youth Risk Behavior Survey Questionnaire Am. J. Epidemiol 1995 141 575 580 7900725 10.1093/oxfordjournals.aje.a117473

